# FoxP3 Tregs Response to Sublingual Allergen Specific Immunotherapy in Children Depends on the Manifestation of Allergy

**DOI:** 10.1155/2015/731381

**Published:** 2015-09-20

**Authors:** Anna Stelmaszczyk-Emmel, Anna Zawadzka-Krajewska, Eliza Głodkowska-Mrówka, Urszula Demkow

**Affiliations:** ^1^Department of Laboratory Diagnostics and Clinical Immunology of Developmental Age, Medical University of Warsaw, Poland; ^2^Department of Pediatric Pneumonology and Allergology, Medical University of Warsaw, Poland

## Abstract

Over the last decades allergic diseases has become a major health problem worldwide. The only specific treatment to date is allergen specific immunotherapy (ASIT). Although it was shown that ASIT generates allergen-tolerant T cells, detailed mechanism underlying its activity is still unclear and there is no reliable method to monitor its effectiveness. The aim of our study was to evaluate ASIT influence on the frequency of forkhead box P3 (FoxP3) Tregs in allergic children with various clinical manifestations. The relative number of FoxP3 Tregs in 32 blood samples from allergic children at baseline and/or after 1 year of ASIT was assessed by flow cytometry. In the entire studied group, the percentage of FoxP3 Tregs did not increase 1 year after ASIT. Nevertheless, the percentage of FoxP3 Tregs after ASIT significantly increased in children with respiratory allergy (conjunctivitis, asthma, and rhinitis) coexisting with nonrespiratory manifestations (food allergy and/or atopic dermatitis), whereas, in patients with respiratory allergy only, the percentage of FoxP3 Tregs decreased. To the best of our knowledge, this is the first report showing various differential FoxP3 Tregs response to ASIT in allergic children. FoxP3 Tregs number could be useful in treatment monitoring. Further studies are warranted to confirm these observations.

## 1. Introduction

Over the last few decades the prevalence of allergic diseases has dramatically increased. According to European Federation of Allergy and Airways Diseases Patients' Association (EFA) approximately 113 million people in Europe suffer from allergic rhinitis and 68 million suffer from allergic asthma. In total, respiratory allergies affect 20–30% of European population and the number of affected individuals is growing [1].

Allergic diseases are caused by a complex, both innate and adaptive immune response to natural environmental allergens, with T helper type 2 (Th2) cells and allergen specific IgE predominance. Typically, allergic diseases are characterized by inflammatory reaction associated with increased production of Th2 cytokines in response to relatively benign environmental antigens (allergens) [2–4].

Allergen specific immunotherapy (ASIT) is so far the only specific treatment of allergic disorders with a potential to modify the course of the disease and is considered the most effective therapeutic approach for deregulated immune response towards allergens, by enhancing immune tolerance mechanisms. The main aim of immunotherapy is the generation of allergen nonresponsive or tolerant T cells in sensitized patients and downregulation of predominant T cell- and IgE-mediated immune response. Multiple studies have shown that ASIT modifies the function of monocytes, B cells, and T cells, as well as basophils, eosinophils, and mast cells count [3, 5–7].

At the T cell level, ASIT reduces allergen specific T cell proliferation and tissue Th2 cytokine production, increases tissue Th1 cytokine release, and induces functional Tregs. Generation of Tregs is an important immunomodulatory mechanism of ASIT as Tregs potently suppress proliferative and cytokine responses to allergens. There are two important subsets of Tregs involved in response to ASIT: thymic-derived CD4+CD25++ forkhead box P3 (FoxP3) Tregs and peripherally derived Tregs (nomenclature according to the recommendations given by Abbas et al.) [8]. Both populations have distinct phenotypes and modes of action [3, 6, 7, 9].

The precise mechanism of allergen specific immunotherapy is unknown; however, both subcutaneous and sublingual ASIT primarily affect the regional antigen-presenting cells, namely, the local dendritic cells subset at the place of administration and in draining lymph nodes. Dendritic cells induce Tregs (CD4+CD25+FoxP3+) and IL-10 producing T cells. Tregs may suppress allergen-induced immune responses in several ways. They utilize multiple inhibitory mediators downregulating the immune response, that is, the generation of antigen-presenting dendritic cells and development of IL-10-producing dendritic cells. Finally, Tregs inhibit Th2 cells function, which can no longer provide cytokines such as IL-3, IL-4, IL-5, IL-9, and IL-13. These cytokines are required for the differentiation, survival, and activity of mast cells, basophils, eosinophils, and mucus producing cells involved in allergic processes and for the tissue homing of Th2 cells [3, 5–7]. It is well proven that Tregs number correlates with the severity of an allergic disease and fluctuates accordingly to remissions and exacerbations.

Majority of allergic patients suffer from respiratory allergy (allergic rhinitis (AR), allergic conjunctivitis (AC), and asthma (CA)). In some patients food allergy (FA) or atopic dermatitis (AD) cooccurs. Recently, a number of reports highlighted the role of Tregs in the course of specific immunotherapy, but, to our knowledge, no study to date directly investigated the relation between Tregs and clinical manifestations of an allergic disease.

We have previously demonstrated that, at the moment of allergy diagnosis, patients with various clinical manifestations of allergy differed in the percentage of FoxP3 Tregs [10]. In this paper we aimed to test how ASIT influences the frequency of FoxP3 Tregs in allergic children and whether FoxP3 Tregs number differs in patients with various clinical manifestations of the disease (respiratory allergy with or without concomitant FA and/or AD).

## 2. Materials and Methods

### 2.1. Patients

The study involved 21 children with pollen respiratory allergy (diagnosed and treated in the Department of Pediatric Pneumonology and Allergology, Medical University of Warsaw, Poland). The diagnosis of allergic rhinitis was based on a typical history of allergic symptoms such as rhinorrhea, sneezing, nasal obstruction, and pruritus and diagnostic tests results including skin prick tests with a panel of allergens and the measurements of allergen specific IgE in serum [11].  Table 1 shows demographic and clinical characteristics of allergic children before and after one year of ASIT. All patients were polysensitized to outdoor pollen allergens. All patients were treated with sublingual immunotherapy against outdoor pollen allergens (Staloral 300, Stallergenes, France). Sensitizing antigens and ASIT formulas for each patient are specified in Table 2.

For 11 children matching samples obtained before and after ASIT were tested, whereas unpaired samples before or after ASIT were available for 6 and 4 children, respectively. In total, 32 blood samples were analyzed, including 17 and 15 samples obtained before and after one year of ASIT (sublingual immunotherapy, Staloral 300, Stallergenes, France), respectively. In each case 1.0–2.0 mL of heparinized blood was collected from antecubital vein.

The study was approved by the Independent Ethics Committee of the Medical University of Warsaw. The parents gave informed consent for the participation in the study. The study was conducted according to the Declaration of Helsinki.

At the time of blood collection none of the patients was treated with glucocorticoids. All tests were conducted outside of the pollen season and the children did not present any symptoms of active infection at the moment of sampling.

### 2.2. Cells

Peripheral blood mononuclear cells (PBMC) were isolated using a standard Ficoll-Histopaque-1077 (Sigma Aldrich Co., St. Louis, USA) gradient centrifugation according to manufacturer's protocol. Cells' concentration was adjusted to 1 × 10^6^/mL in PBS supplemented with 0.5% inactivated FBS. Viability of PBMC was determined by trypan blue staining and achieved approximately 96–98%.

#### 2.2.1. Analysis of Frequency of FoxP3 Tregs

Freshly isolated 100 *µ*L of 1 × 10^6^/mL PBMC was stained with 5 or 10 *µ*L of monoclonal antibodies (according to manufacturer's instructions, BD-Pharmingen): anti-CD25 PE-Cy7, clone M-A251; anti-CD4 PE-Cy5; anti-CD127 PE. The samples were incubated for 20 minutes in the dark at room temperature. Next, the cells were washed twice in a washing buffer (PBS supplemented with 0.5% inactivated FBS) for 5 minutes, 250 g. FoxP3 intracellular staining was performed according to manufacturer's instructions (BD-Pharmingen). Briefly, cells were incubated 10 minutes in room temperature in the dark with 2 mL of fixation buffer and then washed in washing buffer, centrifuged at 500 g for 5 minutes, and incubated for 30 minutes in 500 *µ*L of permeabilization buffer in room temperature in the dark. Subsequently, the cells were stained with 20 *µ*L anti-FoxP3 monoclonal antibody (Alexa Flour 488, BD-Pharmingen) for 30 minutes in room temperature in the dark and washed twice before the analysis.

#### 2.2.2. Flow Cytometric Analysis

**Figure 1:**
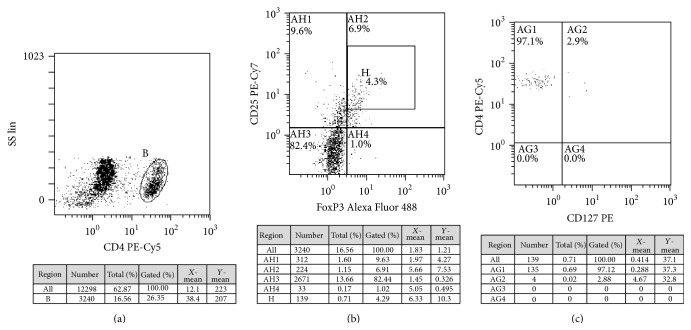
FoxP3 Tregs gating strategy. Example result obtained for a single patient before ASIT. PBMC was stained with combination of surface antigens (anti-CD4, anti-CD25, and anti-CD127) and intracellularly with anti-FoxP3. For analysis, CD4 positive cells from PBMC were gated ((a) dot plot SS versus CD4, gate B) and analyzed for CD25 and FoxP3 expression ((b) dot plot CD25 versus FoxP3 for B gate); cells in H gate were considered Tregs. The expression of CD127 in Tregs population was analyzed ((c) dot plot CD4 versus CD127 for H gate).

In all experiments appropriate isotype controls were included. The samples were evaluated within 24 hours from sampling on Cytomics FC500 flow cytometer (Beckmann Coulter). Tregs in peripheral blood were identified as CD4+CD25+^high^FoxP3+CD127− T cells. It means that CD4CD25^high^ T cells were considered Tregs only when they showed FoxP3 expression and were negative for CD127 expression. The number of Tregs is expressed as a percentage of all CD4+ T cells. Gating strategy is shown in Figure 1.

### 2.3. Statistical Analysis

All of the measured parameters had nonparametric distribution (according to Shapiro-Wilk's criteria), so statistical analysis was performed using nonparametric Mann-Whitney *U* test for independent samples and two-way nonparametric ANOVA with post hoc Fisher's test. To assess the correlations between the results Spearman test was used.

## 3. Results

**Figure 2:**
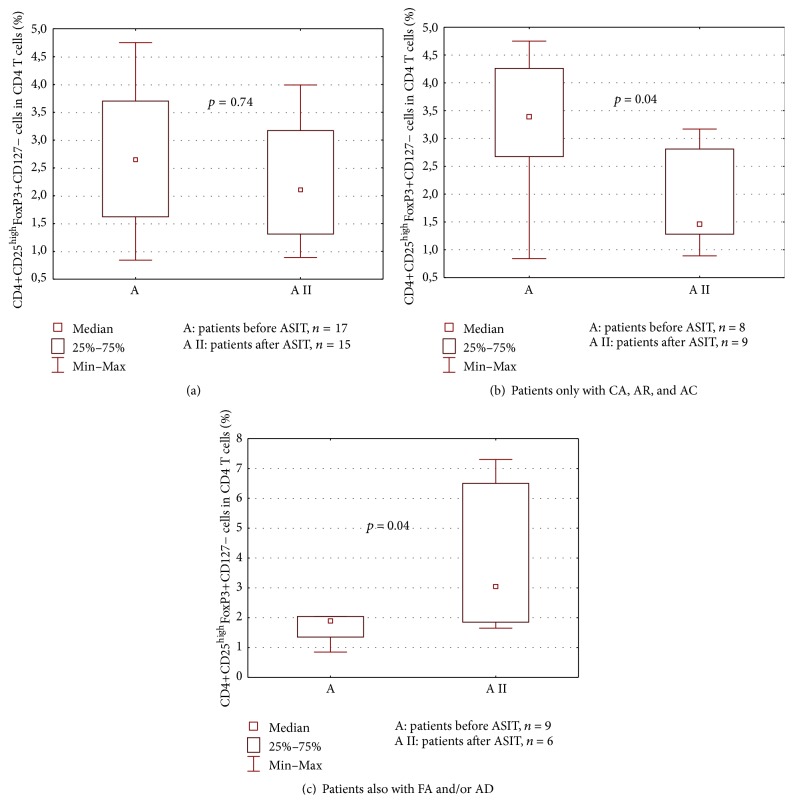
The percentage of CD4+CD25^high^FoxP3+CD127− Tregs in CD4 T cell population in peripheral blood of patients with allergy before ASIT (A) and after ASIT (A II). (a) All patients, (b) first group of patients, only CA, AR, and AC, and (c) second group of patients, with FA and/or AD.

In the whole group of allergic patients, the percentage of FoxP3 Tregs did not change before and after ASIT (median (25 percentile; 75 percentile): 2.65 (1.62; 3.70) and 2.10 (1.31; 3.17), resp.; *p* = 0.74) (Figure 2(a)). The patients were further divided into two groups. Group 1 included patients with allergy limited to respiratory tract, while group 2 consisted of patients with respiratory allergy and concomitant symptoms of AD and/or FA. Significant decrease in the percentage of Tregs was observed in group 1 before and after ASIT (median (25 percentile; 75 percentile): 3.39 (2.67; 4.26) and 1.46 (1.28; 2.81), resp.; *p* = 0.04). Oppositely, in group 2, significant increase in the percentage of FoxP3 Tregs after ASIT was demonstrated (median (25 percentile; 75 percentile): 1.89 (1.35; 2.04) and 3.05 (1.85; 6.5), resp.; *p* = 0.04) (Figures 2(b) and 2(c), resp.).

Significantly lower percentages of Tregs in patients with symptoms of AD and/or FA than in patients free from those clinical presentations before ASIT (median (25 percentile; 75 percentile): 1.89 (1.35; 2.04) and 3.39 (2.67; 4.26), resp.; *p* = 0.04) were observed. The same comparison after ASIT showed opposite results; patients with additional clinical manifestations had significantly higher percentages of Tregs (median (25 percentile; 75 percentile): 3.04 (1.85; 6,50) and 1.46 (1.28; 2.81); *p* = 0.04).

Relative fluorescence intensity (RFI) of FoxP3 expression was also compared. RFI was calculated according to Dechant et al. using the following formula: experimental mean fluorescence intensity (MFI)/MFI with isotype control antibody [12]. We observed that median RFI of FoxP3 expression in patients before ASIT was higher in comparison to patients after ASIT (median (25 percentile; 75 percentile): 11.26 (9.69; 13.16) and 7.65 (4.16; 12.87), resp.), but the difference was statistically insignificant.

The comparison of CD4+FoxP3+ T cells and CD4+CD25+ T cells in subgroups of patients before and after ASIT did not reveal any differences. Additionally, the percentage of lymphocytes, the number of lymphocytes/*µ*L, and the number of WBC/*µ*L did not significantly differ between both studied groups.

## 4. Discussion

Although the obtained results, in the entire examined group of patients, did not bring any unexpected conclusions, the analysis of subgroups of patients with the presence or absence of extra-respiratory manifestations revealed significant differences in the number of FoxP3 Tregs. Accordingly, this observation pinpoints the differences in immunological response to ASIT, depending on clinical manifestations of atopic allergy. Recently we have shown that the percentage of FoxP3 Tregs at baseline does not depend on the nature or number of disease-causing allergens in specific subgroups of patients; however, the frequency of those cells differs depending on the disease locations [10]. The same observation was confirmed in the current study. Similar relation persisted after one year of immunotherapy.

Interestingly, we observed that specific immunotherapy had stronger impact on the frequency of FoxP3 Tregs in peripheral blood in patients with more extensive allergic disease and lower level of Tregs at baseline. In addition, the patients with FD and/or AD have statistically lower RFI for FoxP3. It means that although the number of cells increased, the actual expression of transcription factor in each cell was lower. Some authors correlated MFI or RFI of FoxP3 with Tregs function [13]. After allergen provocation Thunberg et al. showed higher MFI for FoxP3 in BAL fluid-derived cells, but not in blood [13].

As previously mentioned, after one year of ASIT no changes in the percentage of FoxP3 Tregs in peripheral blood in the entire examined group of allergic children were observed. This observation corresponds with other authors findings. The majority of authors did not demonstrate any alteration in the number of FoxP3 Tregs (previously called natural Tregs) after immunotherapy or allergen provocation [13–17], whereas others showed that FoxP3 Tregs population is increased after ASIT [18–21]. To fully understand these discrepancies the two issues have to be taken into consideration: the definition of “Tregs” and methods used to identify this minor population of cells. Those inconsistencies between different studies make them not fully comparable.

The majority of authors unanimously claim that the efficacy of ASIT does not depend on FoxP3 Tregs, but on another population of Tregs, namely, IL-10 producing Tregs. They postulated that the number of IL-10 producing T cells may be considered a biomarker for monitoring of response to ASIT [22]. Increased number of IL-10 producing Tregs after ASIT was shown in both allergic adults and children [18, 22–26]. We also attempted to analyze IL-10 in examined patients; however, the concentrations of IL-10 in blood samples from allergic children were below the detection level of Cytometric Bead Array (BD Biosciences) and it was not possible to draw any conclusions from the obtained results (data not shown).

Furthermore the discrepancies between the studies can be due to differing observation period, possible exposition to different allergens, and type of medication used. Our study was performed after one year (including single pollen season) in the same, relatively small geographical area, so the contact with environmental allergens was similar for all enrolled patients. To further diminish the potential influence of environmental allergens, the blood for the analysis was not collected during pollen season. In addition, to further limit the influence of confounding factors, the same type of medication was applied to all patients. Finally the differences between studies could be attributed to the variety of clinical material used to establish immune response to ASIT. We had the opportunity to test peripheral blood, but some authors used different specimens such as sublingual epithelium, BAL fluid, or nasal mucosa [13, 27, 28]. As it was shown by Thunberg et al., the cells obtained from peripheral blood are not always indicative for the target organ [13]. These authors contrasted peripheral blood and BAL fluid findings after allergen provocation and demonstrated increased expression of FoxP3 in BAL fluid without any respective alterations in peripheral blood. Scadding et al. demonstrated that the number of FoxP3 Tregs was increased in the sublingual epithelium following grass pollen SLIT [27], whereas Radulovic et al. showed consistent relation in the nasal mucosa [28].

In view of a large number of studies FoxP3 Tregs directly influence various immune cells which participate in the development of allergic reactions including mast cells, eosinophils, basophils, Th2, and B cells. The elevation in Tregs number after ASIT contributes to deactivation of those cells in skin, nose, eye, and mucosal tissues. As a consequence, severity of patients symptoms and their quality of life can improve. It is particularly important for patients with multiorgan involvement. It is well proven that ASIT is effective in long-term observation period and can prevent new allergen sensitization. In addition, some authors showed that ASIT can also modulate the course of FA and AD [7].

Allergic diseases are not the only conditions that improve as a result of increased activity of Tregs. As Tregs suppress excessive immune system activation and promote immunologic tolerance, some clinical trials attempted Tregs immunotherapy in type 1 diabetes or in graft versus host disease [4, 29–31]. Accordingly allergic patients could potentially benefit from Tregs-targeted immunotherapy. On the other hand, we have shown that in a significant group of patients ASIT does not increase the number of potentially beneficial FoxP3 Tregs, while it is clinically effective. Further studies are warranted to thoroughly describe the mechanism of action of ASIT in this group of patients.

The main limitation of our study was that we were not able to perform the entire analysis in matching samples, because as much as 6 of 17 patients were lost to follow-up. Hence we included additional 4 patients who fulfilled inclusion criteria and were tested after one year of ASIT. Accordingly we used nonparametric test for independent samples for analysis.

In conclusion, we analyzed FoxP3 Tregs expression in allergic patients undergoing ASIT and we showed differing pattern of Tregs response in patients with various clinical manifestations. The immunological reactions which caused elevation of FoxP3 Tregs in group 2 are not obvious and require further studies. The understanding of the mechanisms underlying immunoregulation in different groups of patients may lead to development of more effective treatment of allergic diseases.

## Figures and Tables

**Table 1:** Demographic and clinical characteristics of children with allergy before and one year after ASIT.

	Before ASIT	After ASIT
Number	17	15
Males	15	15
Females	2	0
Age (mean ±SD years)	7.9 ± 2.65	8.9 ± 2.86
Clinical symptoms		
Group 1	8	9
Group 2	9	6
WBC (average ±SD cells/*µ*L)	7817 ± 2427.4	6646 ± 1482.7
Median	8300	6100
Lymphocytes (average ±SD cells/*µ*L)	3247 ± 982.4	2827 ± 677.7
Median	3300	2700
Lymphocytes (average ±SD %)	41.7 ± 11.62	43.0 ± 5.89
Median	43	42
Eosinophils (average ±SD cells/*µ*L)	347 ± 373.3	304 ± 109,1
Median	277	324
Eosinophils (average ±SD %)	4.6 ± 3.61	4.7 ± 1.84
Median	3.8	4.1
sIgE (kU/L, scale 0–6)	>3 class	

Group 1: patients with respiratory allergy: allergic rhinitis (AR), allergic conjunctivitis (AC), and asthma (CA).

Group 2: patients with respiratory allergy and food allergy (FA) or atopic dermatitis (AD).

**Table 2:** Sensitizing antigens and ASIT formulation.

Patient	Number and type of outdoor allergens	Number and type of indoor allergens	ASIT formulation (Staloral 300)
1	2, grass and rye pollen	0	Grass pollen 100%
2	2, grass and rye pollen	0	Grass pollen 80%, rye pollen 20%
3	3, birch, alder, and hazel pollen	0	Birch pollen 35%, alder pollen 30%, and hazel pollen 35%
4	6, grass, rye, mugwort, birch, alder, and hazel pollen	2, *D. pteronyssinus*, *D. farinae*	Grass pollen 60%, rye pollen 40%
5	3, grass, birch, and alder pollen	1, dog allergens	Grass pollen 100%
6	7, grass, rye, mugwort, plantain, birch, alder, and hazel pollen	1, dog allergens	Grass pollen 60%, rye pollen 40%
7	6, birch, alder, hazel, grass, rye, and mugwort pollen	2, dog allergens, cat allergens	Birch pollen 35%, alder pollen 30%, and hazel pollen 35%
8	2, grass and rye pollen	0	Grass pollen 80%, rye pollen 20%
9	5, grass, rye, birch, alder, and hazel pollen	0	Grass pollen 60%, rye pollen 40%
10	6, birch, alder, hazel, rye, mugwort, and grass pollen	0	Birch pollen 35%, alder pollen 30%, and hazel pollen 35%
11	3, grass, rye, and birch pollen	4, *D. pteronyssinus, D. farinae*, dog allergens, and cat allergens	Grass pollen 80%, rye pollen 20%
12	3, birch, alder, and hazel pollen	0	Birch pollen 35%, alder pollen 30%, and hazel pollen 35%
13	7, grass, rye, birch, alder, hazel, mugwort, and plantain pollen	0	Grass pollen 80%, rye pollen 20%
14	2, grass and rye pollen	0	Grass pollen 80%, rye pollen 20%
15	3, birch, alder, and hazel pollen	1, cat allergens	Birch pollen 35%, alder pollen 30%, and hazel pollen 35%
16	3, grass, rye, and birch pollen	0	Grass pollen 60%, rye pollen 40%
17	2, grass and rye pollen	0	Grass pollen 60%, rye pollen 40%
18	2, grass and rye pollen	0	Grass pollen 80%, rye pollen 20%
19	3, grass, rye, and mugwort pollen	0	Grass pollen 60%, rye pollen 40%
20	2, grass and rye pollen	0	Grass pollen 60%, rye pollen 40%
21	5, birch, alder, hazel, grass, and rye pollen	2, *D. pteronyssinus*, *D. farinae*	Birch pollen 35%, alder pollen 30%, and hazel pollen 35%
